# Regulating Immunogenicity and Tolerogenicity of Bone Marrow-Derived Dendritic Cells through Modulation of Cell Surface Glycosylation by Dexamethasone Treatment

**DOI:** 10.3389/fimmu.2017.01427

**Published:** 2017-10-30

**Authors:** Kevin Lynch, Oliver Treacy, Jared Q. Gerlach, Heidi Annuk, Paul Lohan, Joana Cabral, Lokesh Joshi, Aideen E. Ryan, Thomas Ritter

**Affiliations:** ^1^School of Medicine, Regenerative Medicine Institute (REMEDI), National University of Ireland Galway, Galway, Ireland; ^2^Glycoscience Group, NCBES National Centre for Biomedical Engineering Science, National University of Ireland Galway, Galway, Ireland; ^3^Discipline of Pharmacology and Therapeutics, School of Medicine, National University of Ireland, Galway, Ireland

**Keywords:** tolerogenic dendritic cells, glycosylation, dexamethasone, immunogenicity, tolerogenicity, sialic acid, autoimmunity, cell therapy

## Abstract

Dendritic cellular therapies and dendritic cell vaccines show promise for the treatment of autoimmune diseases, the prolongation of graft survival in transplantation, and in educating the immune system to fight cancers. Cell surface glycosylation plays a crucial role in the cell–cell interaction, uptake of antigens, migration, and homing of DCs. Glycosylation is known to change with environment and the functional state of DCs. Tolerogenic DCs (tDCs) are commonly generated using corticosteroids including dexamethasone, however, to date, little is known on how corticosteroid treatment alters glycosylation and what functional consequences this may have. Here, we present a comprehensive profile of rat bone marrow-derived dendritic cells, examining their cell surface glycosylation profile before and after Dexa treatment as resolved by both lectin microarrays and lectin-coupled flow cytometry. We further examine the functional consequences of altering cell surface glycosylation on immunogenicity and tolerogenicity of DCs. Dexa treatment of rat DCs leads to profoundly reduced expression of markers of immunogenicity (MHC I/II, CD80, CD86) and pro-inflammatory molecules (IL-6, IL-12p40, inducible nitric oxide synthase) indicating a tolerogenic phenotype. Moreover, by comprehensive lectin microarray profiling and flow cytometry analysis, we show that sialic acid (Sia) is significantly upregulated on tDCs after Dexa treatment, and that this may play a vital role in the therapeutic attributes of these cells. Interestingly, removal of Sia by neuraminidase treatment increases the immunogenicity of immature DCs and also leads to increased expression of pro-inflammatory cytokines while tDCs are moderately protected from this increase in immunogenicity. These findings may have important implications in strategies aimed at increasing tolerogenicity where it is advantageous to reduce immune activation over prolonged periods. These findings are also relevant in therapeutic strategies aimed at increasing the immunogenicity of cells, for example, in the context of tumor specific immunotherapies.

## Introduction

Dendritic cells are professional antigen-presenting cells, which are a component of the innate immune system which induce adaptive immune responses (1). Dendritic cells (DCs) were first described by Steinman and Cohn in 1973 (2) and were subsequently identified to be potent activators of the immune system when employed in mixed lymphocyte reactions (MLRs) (3). DCs are a heterogeneous population classified in different subsets dependent on the origin (4). DCs have been extensively investigated for potential use as a cellular therapy due to their ability to maintain peripheral tolerance, which is of importance in the field of transplantation and autoimmunity. Since mature DCs are potent activators of the T-cell responses, pharmacological approaches have been used to maintain DCs in a maturation resistant state (5–7). The glucocorticoid dexamethasone (Dexa) has been widely used in this context (8–11). Glucocorticoids are potent immunosuppressive drugs that are used in clinical regimens to treat both Th1- and Th2-mediated inflammatory diseases including allograft rejection (12). Dexa is known to exert potent effects on many immune cells including DCs (8, 13). It has been consistently described in the literature that Dexa has inhibitory effects on the development of immature DCs (iDCs) (5, 8, 12, 14), and that it also impairs lipopolysaccharide (LPS) (TLR4) stimulation of DCs, which would otherwise lead to their maturation (mDCs) (15–17). In addition to this, Dexa-treated DCs have a reduced capacity to activate naïve T lymphocytes by interfering with Signals 1–3 important for T-cell activation (17).

In the context of transplantation, preclinical experiments suggested the potential therapeutic use of both donor and recipient-derived tolerogenic DCs to prevent organ graft rejection (18). In a rat model, we have recently shown that pretreatment of donor DCs with Dexa *ex vivo* prevents the maturation of DCs and prolongs rat corneal allograft survival upon injection in corneal transplant recipients (13). However, the mechanisms of how tolerogenic DCs engage with other immune cells and exert their immunomodulatory effects are not completely understood. Despite this, tolerogenic DCs have been already tested in humans suffering from various diseases. As of this writing, there are currently eight tolerogenic DC cell therapies listed in Phase I/II clinical trials for treatment of autoimmune disease and graft rejection (https://clinicaltrials.gov. September 2017, search for key words tolerogenic DCs), which highlights the importance and urgency of understanding the mechanisms associated with the therapeutic effect.

Glycosylation is one of the most vital and frequent forms of posttranslational modification and is involved in the function of many immune associated molecules. Some of the functions of glycosylation include, but are not limited to, protein folding and molecular trafficking to the cell surface (19–23). Glycosylation has also been implicated in the stability of proteins and protection from proteolysis (24). All immune cells are coated by a glycocalyx composed of a complex assortment of oligosaccharides (glycans), of which one frequent terminal component is sialic acid (Sia). Sias are a broad family of negatively charged, 9-carbon monosaccharides that are exposed to the cellular microenvironment and are involved in communication and in cellular defense (25). It has been reported that a typical somatic cell surface presents millions of Sia molecules (26) and also that they have long been noted to be important in immune cell behavior (27). It has been suggested that Sias can play important roles in both acting as a recognizable molecule for cellular interactions but also as a biological shield preventing receptors on cells recognizing their ligands (28). Large amounts of Sias on the cell surface of immune cells will result in an overall negative charge, which can have biophysical effects, such as the repulsion of cells from each other and subsequently disrupting cellular interactions (29).

Since immune cell interactions form the basis of immune responses, glycosylation is, therefore, likely to play a major role in dictating these responses. However, there is a significant knowledge gap as to how glycosylation modulates immune responses. Currently, little information exists on how DC glycosylation patterns change after Dexa treatment. Here, we present a comprehensive profile of bone marrow-derived DCs (BMDCs), examining their cell surface glycosylation before and after Dexa treatment as resolved by both lectin microarrays and lectin-coupled flow cytometry.

In this work, the composition of the glycocalyx of both iDCs and tolerogenic DCs (tDCs) was altered using neuraminidase (sialidase) treatment and the functional consequences in immunogenicity and inhibition of T-cell proliferation were observed. We show that Sia is upregulated on tDCs contributing to the tolerogenic state of tDCs. However, removal of Sia leads to increased stimulatory activity of iDCs leading to enhanced T-cell activation and proliferation. These findings have important implications in strategies aimed at increasing tolerogenicity where it is advantageous to reduce immune activation over prolonged periods. These findings are also relevant in therapeutic strategies aimed at increasing the immunogenicity of cells, for example, in the context tumor specific immunotherapies.

## Materials and Methods

### Animals

All animals used in experiments were accommodated in an accredited animal housing facility under a license granted by the Department of Health, Ireland, and were approved by the Animals Ethics Committee of the National University of Ireland, Galway. Bone marrow used in the generation of BMDCs was isolated from male Dark Agouti (DA, RT-1^avl^) rats at 8–14 weeks of age. For the allogeneic MLRs, male Lewis (LEW, RT-1^l^) rats served as a source of lymphocytes, isolated from both the cervical and mesenteric lymph nodes and spleen. DA and LEW rats were obtained from Harlan Laboratories (Bicester, UK).

### Isolation and Generation of iDCs and tDCs

Immature DCs were generated using an adapted version of the protocol, which has been previously described (13) (Figure S1 in Supplementary Material). Briefly, on day 0, male DA rats of the specified age were sacrificed and the tibia and femur were surgically removed postmortem. The epiphyses were cut and the bone marrow was flushed from the long bones with a syringe/needle combination. The erythrocytes were removed from the suspension by lysis using a standard red blood cell lysis buffer (Sigma-Aldrich, Dublin, Ireland). After erythrocyte lysis, the cells were washed in RPMI-1640 (Gibco, Grand Island, NY, USA) medium supplemented with 10% heat-inactivated fetal bovine serum (FBS), 2 mmol/L l-glutamine, 100 mmol/L nonessential amino acids, 1 mmol/L sodium pyruvate, 100 U/mL penicillin, 100 µg/mL streptomycin, and 55 µmol/L 2-β-mercaptoethanol (2β-ME) (Gibco). Cells were resuspended at a concentration of 1.5 × 10^6^/mL and plated at a concentration of 4.5 × 10^6^ per well of a 6-well plate. The culture medium was supplemented with 5 ng/mL rat granulocyte-macrophage colony-stimulating factor (GM-CSF) (Invitrogen, Paisley, UK) and 5 ng/mL rat IL-4 (Peprotech EC, London, UK). Cells were incubated under standard cell culture conditions (37°C at 5% CO_2_) and, on the third day of culture, half of the medium from each well was harvested and cells were resuspended in fresh medium supplemented with rat GM-CSF and IL-4 and added back into the culture. On the fifth day, the supernatant was exchanged with fresh supplemented growth medium to remove dead granulocytes and lymphocytes. In experiments requiring tDCs, Dexa (Sigma-Aldrich) was added to the culture at 10^−6^ mol/L at this point. On the seventh day of culture, half of the medium was again removed and replaced with fresh supplemented medium (Dexa was added as required). To generate mDCs, LPS (1 µg/mL; Sigma-Aldrich) was added 24 h before the cells were cultured. Cultures were maintained until day 10 and then gently pipetted off the bottom of the wells for the *in vitro* assays.

### Neuraminidase Treatment

To produce neuraminidase-treated iDCs and tDCs (niDCs and ntDCs), BMDCs were harvested on day 10 of culture and 2 × 10^5^/mL were treated with 400 U/mL of recombinant *Clostridium perfringens* neuraminidase (P0720S, New England Biolabs, Ipswitch, MA, USA) in phosphate buffered saline (PBS) supplemented with 1 mM MgCl_2_ (Sigma-Aldrich), 1 mM CaCl_2_ (Sigma-Aldrich), and 1% bovine serum albumin (Sigma) for 90 min at 37°C.

### RNA-Isolation and RT-PCR

RNA was exacted from iDCs, tDCs, mDCs, niDCs, and ntDCs on day 10 using Bioline Isolate II RNA mini kits according to manufacturer’s protocols. All cDNA was produced using RevertAid^TM^ H Minus Reverse Transcriptase (Thermo Fisher Scientific, MA, USA) with random primers. For primer sequences of GAPDH, TNF-α, IL-12p40, inducible nitric oxide synthase (iNOS) IL-10, IDO, IL-6, and IL-1β, see Table S1 in Supplementary Material. All samples were normalized to expression of the house-keeping gene GAPDH and made relative to iDCs. All quantitative real-time PCR was performed according to the standard program using a real-time PCR system (StepOne Plus, Applied Biosystems, Thermo Fisher Scientific).

### Flow Cytometry

Cells were characterized by flow cytometry using the monoclonal antibodies (mAbs) CD11b/c-APC, CD80-PE, CD86-PE, MHCI-FITC, and MHCII-PE (BioLegend, San Diego, CA, USA). For analysis of the glycocalyx, lectins from *Maackia amurensis* (MAL II, indicating α2-3 Sia) and *Sambucus nigra* (SNA-I, indicating α2-6 Sia) were used (Vector Labs). Lectins were biotin conjugated. PE-streptavidin was used for detection. Negative controls for non-specific fluorescence were used, these consisted of PE-streptavidin staining solutions in the absence of the lectin conjugated to biotin. Lectins were prepared in lectin staining buffer (PBS containing 1% FBS, 1 mmol/L CaCl_2_, and 2 mmol/L MgCl_2_) and resuspended in FACS buffer (PBS containing 2% fetal calf serum and 0.01% NaN_3_, all from Sigma-Aldrich) before analysis using a FACS Canto II (BD Biosciences, Oxford, UK).

For analysis of the assays involving lymphocytes from the lymph nodes and spleen, the following mAbs were used CD3/PE, CD8/PE-Cy7, CD4/APC (BioLegend), and CD25/FITC (eBioscience, San Diego, CA, USA). Prior to staining, cells were washed with FACS buffer. mAbs were diluted in 50 µL FACS buffer, added to the cells, and incubated for 15 min at 4°C. To remove any unbound antibodies, the cells were washed three times with FACS buffer. The cells were then filtered through a nylon mesh (40 µm) before analysis in the cytometer.

### Mixed Lymphocyte Reaction/T Cell Proliferation Assays

Lymphocytes were isolated from the spleen and lymph nodes of LEW rats. T cells were washed with phosphate-buffered saline and stained in prewarmed (37°C) CellTrace™ Violet (CTV) phosphate-buffered saline staining solution (Invitrogen, Carlsbad, CA, USA) as per manufacturer’s instructions. 2 × 10^5^ CTV-stained T cells were stimulated at a 1:1 ratio with anti-rCD3/anti-rCD28-labeled beads in supplemented RPMI 1640 media. Assays were incubated at various BMDC: T-cell ratios in a humidified incubator for 4/5 days at 37°C following which T-cell proliferation and CD4 and CD8 expression were assayed by flow cytometry (mAbs CD4-APC and CD8α-PE-Cy7; Biolegend). T-cell proliferation, activation, and differentiation were analyzed using a FACS Canto II.

### Membrane Protein Extraction and Labeling

Membrane proteins were extracted from iDCs, tDCs, niDCs, and ntDCs using a commercial protein extraction kit (Mem-Per^®^, Thermo Fisher Scientific). Proteins recovered from 10^6^ cells were labeled with 100 µg (10 mg/mL in DMSO) Alexa Fluor^®^ succinimidyl ester 555 dye (Thermo Fisher Scientific) as per the manufacturer’s instructions. Labeled protein was separated from unconjugated dye with Bio-Gel^®^ P6 (Bio-Rad Laboratories, Dublin, Ireland).

### Lectin Microarray Construction and Sample Interrogation

Lectin microarrays were constructed essentially as described previously in Ref. (30). Forty-four lectins (Table S2 in Supplementary Material) sourced from multiple vendors were diluted to 0.5 mg/mL in PBS supplemented with 1 mM of respective haptenic sugar to maintain binding site integrity (see Table S2 in Supplementary Material) and printed on Nexterion^®^ H (Schott, Mainz, Germany) functionalized glass substrates using a sciFLEXARRAYER S3 non-contact spotter (Scienion, Berlin, Germany). During printing, relative humidity and temperature were maintained at 62% (±2%) and 20°C, respectively. Following printing, slides were incubated in a humidity chamber overnight at 20°C to ensure completion of covalent conjugation. Unoccupied functional groups were deactivated by 1 h incubation with 100 mM ethanolamine in 50 mM sodium borate, pH 8. Finished slides were washed with PBS with 0.05% Tween-20 (PBS-T) three times for 3 min and once with PBS for 3 min, centrifuged dry (450 × *g*, 5 min), and stored at 4°C with desiccant until use.

Labeled cellular proteins were incubated with finished microarrays following extensive optimization as described in Ref. (30). All processes were carried out with limited light exposure. Samples were applied to microarrays using an 8-well gasket slide and incubation cassette system (Agilent Technologies, Cork, Ireland). 70 µL of each labeled glycoprotein at 0.5 mg/mL, in incubation buffer [TBS-T; Tris-buffered saline (TBS; 20 mM Tris–HCl, 100 mM NaCl, pH 7.2, supplemented with 1 mM CaCl_2_, and 1 mM MgCl_2_) with 0.05% Tween^®^-20], was applied to each well of the gasket. A total of 18 technical replicates were carried out for iDC and tDC profiling (encompassing samples of five biological replicates). Each microarray slide was loaded into a cassette with an accompanying gasket slide and placed in a rotating incubation oven (23°C, approximately 4 rpm) for 1 h. Incubation cassettes were disassembled under TBS-T, and microarrays were washed in a Coplin jar twice in TBS-T for 2 min each and once with TBS for 2 min. Microarrays were dried by centrifugation (450 × *g*) and imaged immediately using an Agilent G2505B microarray scanner at 5 µm resolution (532 nm laser, 100% laser power, 90% PMT).

### Microarray Data Extraction and Analysis

Data extraction and analysis was performed essentially as previously described (30, 31). In brief, raw intensity values were extracted from high-resolution *.tif files using GenePix Pro v6.1.0.4 (Molecular Devices, Berkshire, UK) and a proprietary *.gal file (containing feature spot addresses and identities) using adaptive diameter (70–130%) circular alignment based on 230 mm features. Numerical data were exported as text to Excel (Version 2010, Microsoft, Dublin, Ireland). Local background-corrected median feature intensity data (F543median-B543) was analyzed. The median value, derived from data from six replicate spots per subarray, was handled as a single data point for graphical and statistical analyses.

Lectin microarray intensity values were normalized to the median total intensity value for all features across all subarrays. The significance of difference between relative intensity data (*p < 0.05, **p < 0.01, ***p < 0.001, ****p < 0.0001) was evaluated for each set of replicates on a lectin-by-lectin basis using a standard Student’s *t*-test (two-tailed, two sample unequal variance). Unsupervised, hierarchical clustering of lectin-binding data was performed with Hierarchical Clustering Explorer v3.0 (http://www.cs.umd.edu/hcil/hce/hce3.html). For clustering analysis, previously, normalized data were imported directly and clustered with the following parameters: no pre-filtering, complete linkage, Euclidean distance. Principal component analysis (PrCA) of previously normalized and pre-filtered data (those lectins which demonstrated *p* < 0.01 or better in the above *t*-tests, 15 in total) was performed using Minitab version 16.1.1 (Minitab, Inc., State College, PA, USA).

### Statistical Analysis

Data were analyzed using the software package FlowJo v10 (Tree Star, Ashland, OR, USA). All data were analyzed with Graphpad Prism V6 software (Graphpad Software, CA, USA) and are expressed as mean ± SEM unless otherwise indicated. Comparisons among multiple groups were made with one-way ANOVAs followed by Tukey’s multiple comparisons test. Data sets with two groups were analyzed using an unpaired *t*-test. Differences were considered statistically significant when *p-*value was <0.05.

## Results

### Dexamethasone Treatment of BMDC Induces a Tolerogenic Phenotype

**Figure 1:**
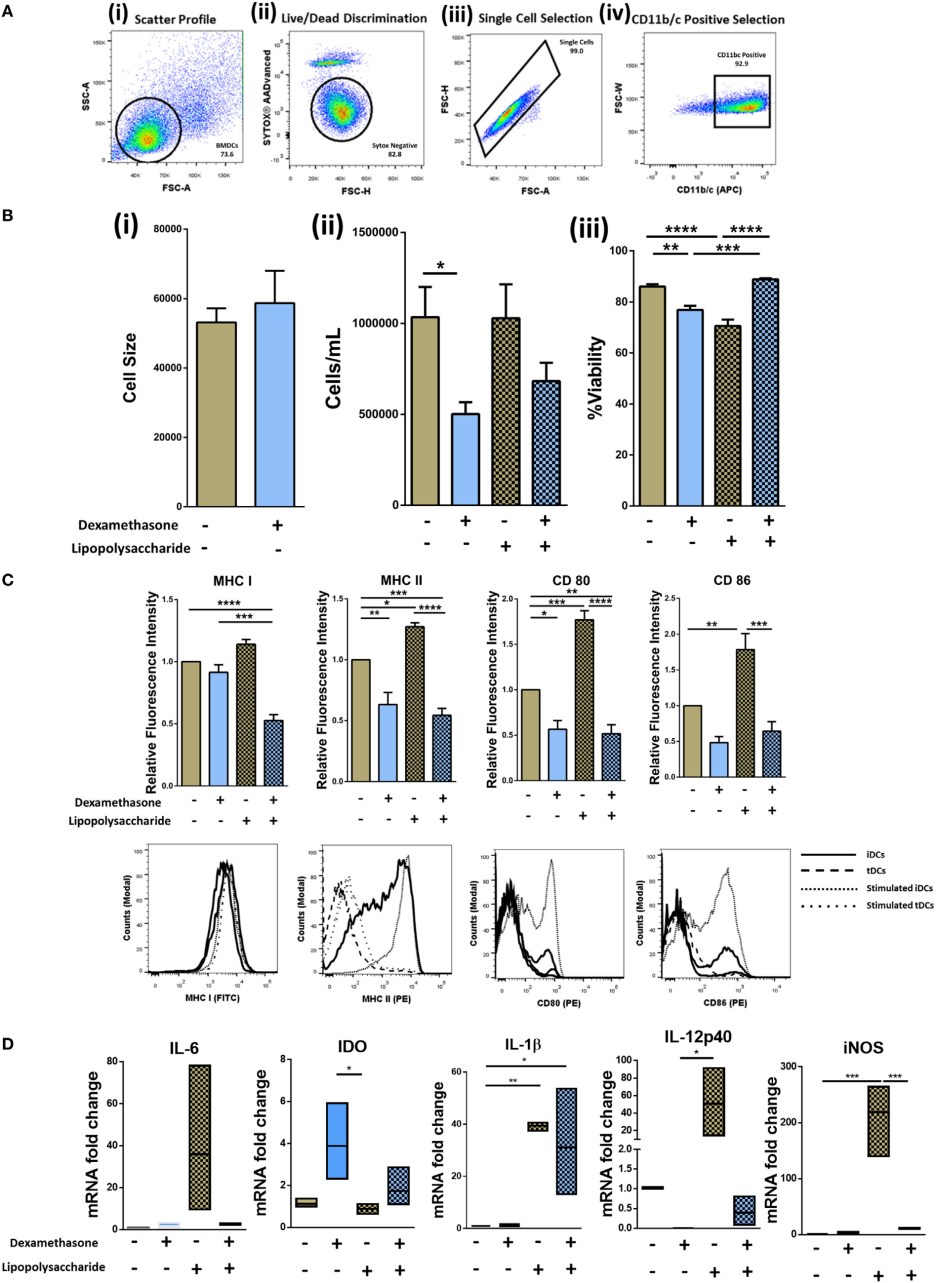
Isolation, generation, and characterization of immature DCs (iDCs), tolerogenic DCs (tDCs), and stimulated DCs (mDCs). Bone marrow was flushed from the femur and tibia of 8- to 14-week-old DA rats and cultured in IL-4 and GM-CSF cultured media for 10 days (Figure S1 in Supplementary Material). **(A)** Representative gating strategy. Cells were selected according to size and granularity (i) followed by live/dead discrimination based on Sytox blue negative cells (live) (ii). After single cell selection (iii), cells were selected by CD11b/c (APC) positivity (iv). **(B)** Changes in cell size (*n* = 3) (i), the number of cells harvested (*n* = 8) (ii), and viability of iDCs to tDCs (*n* = 4) (iii) was compared. **(C)** Both immature DCs (iDCs) and tDCs were analyzed by flow cytometry for their cell surface expression of MHC I (FITC), MHC II (PE), CD 80 (PE) and CD 86 (PE). Representative histograms and bar charts displaying relative fluorescence intensity (RFI) for flow cytometric analysis of DC cell surface. Median fluorescence intensities were established relative to iDCs. **(D)** The mRNA expression of interleukin 6 (IL-6), Indoleamine 2,3-dioxygenase (IDO), interleukin 1 beta (IL-1β), inducible nitric oxide synthase (iNOS), and IL-12p40 was analyzed in iDCs and tDCs. Normalized to GAPDH and fold change relative to iDCs. Error bars: mean ± SEM *p < 0.05, **p < 0.01, ***p < 0.001, ****p < 0.0001 one-way ANOVA, Tukey’s multiple comparisons test.

Dexamethasone treatment of DCs has been reported to generate tolerogenic DCs (tDCs) (32). To generate iDCs, bone marrow was flushed from the long bones of the tibia and femur of DA rats and cultured in medium supplemented with GM-CSF, IL-4, and Dexa (for tDCs) as required (Figure S1A in Supplementary Material). Following isolation, cell surface characterization was performed using flow cytometry by gating on the CD11b/c population (Figure 1A). tDC generation did not result in any significant changes in cell size (Figure 1B, i) but the number of cells harvested from wells that were treated with Dexa was significantly lower than that of wells that were Dexa-free (Figure 1B, ii). This may be due to Dexa-induced apoptosis of the DCs, which has been reported by other groups (33). While lower numbers of cells were obtained from tDC wells, after harvesting and washing of the cells, no significant changes in viability was noted (Figure 1B, iii). We also analyzed the expression levels of the costimulatory molecules CD80/CD86 and the major histocompatibility complex class I and II molecules (MHCI/II) as an indicator of the maturation status of generated iDCs and tDCs (Figure 1C). The expression levels of CD80, CD86, MHC I, and MHC II indicate that the iDCs display a semi-mature phenotype. However, when the cells were treated with Dexa, a significant reduction in the expression level of MHC II was observed with no changes in MHC I (Figure 1C). To mature iDCs or tDC *in vitro*, LPS was added to the cultures (1 µg/mL) for 24 h. A significant increase in both CD80/CD86, MHC I and MHC II was noted. However, tDCs following LPS treatment showed significantly reduced expression levels of CD80/CD86 and MHC I/II molecules compared to stimulated iDCs indicating a phenotype that is maturation resistant. iDC and tDC populations were also assessed for expression of pro- and anti-inflammatory markers with and without Dexa-treatment by qRT-PCR (Figure 1D). Results indicate that LPS stimulation of iDCs leads to an increase in mRNA expression of pro-inflammatory molecules such IL-6, IL-12p40, and iNOS. In contrast, tDCs are less sensitive to TLR4 stimulation compared to mDCs, indicated by no observed increases in IL-6, IL-12-p40, and iNOS after LPS treatment. Higher levels of IDO mRNA, which is known as a marker in tolerogenic cells, is present in LPS-treated tDCs when compared to mDCs. Interestingly, IL-1β mRNA expression does not seem to be regulated by Dexa, as LPS stimulation leads to a profound increase, which cannot be blocked by Dexa. All together these data indicate that Dexa treatment of iDCs leads to the generation of a tolerogenic DC phenotype with reduced expression of markers of immunogenicity and reduced expression of pro-inflammatory molecules but increases in immunoregulatory molecules.

### tDC Generation Modulates the Glycocalyx by Significantly Increasing Levels of α2-6-Linked Sia

Changes in DC glycocalyx after induction of tolerogenic phenotype have not been investigated. To address this knowledge gap, lectin microarray profiling of proteins extracted from the membranes of iDCs and tDCs and lectin-coupled flow cytometry of intact iDCs and tDCs was undertaken.

**Figure 2:**
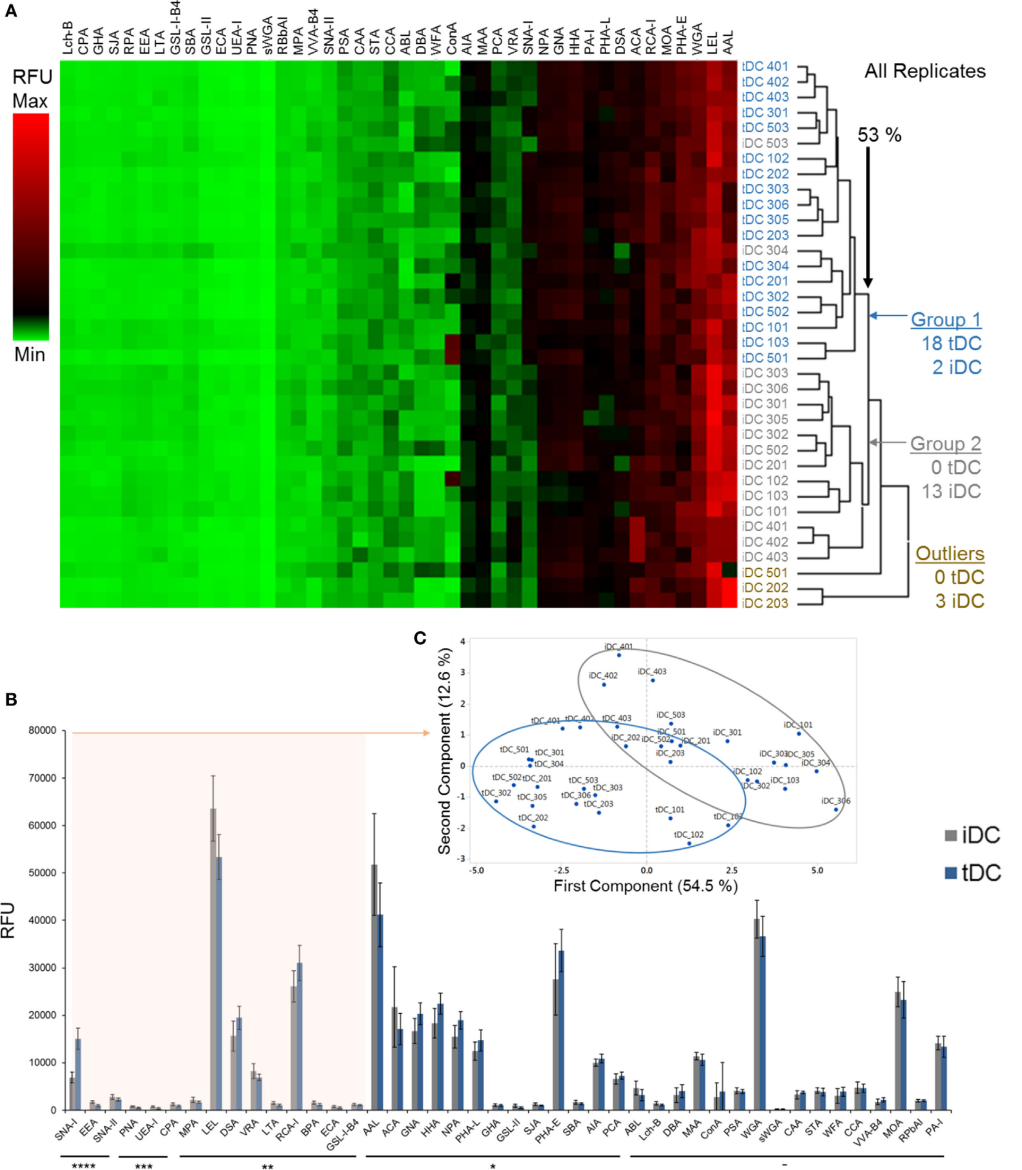

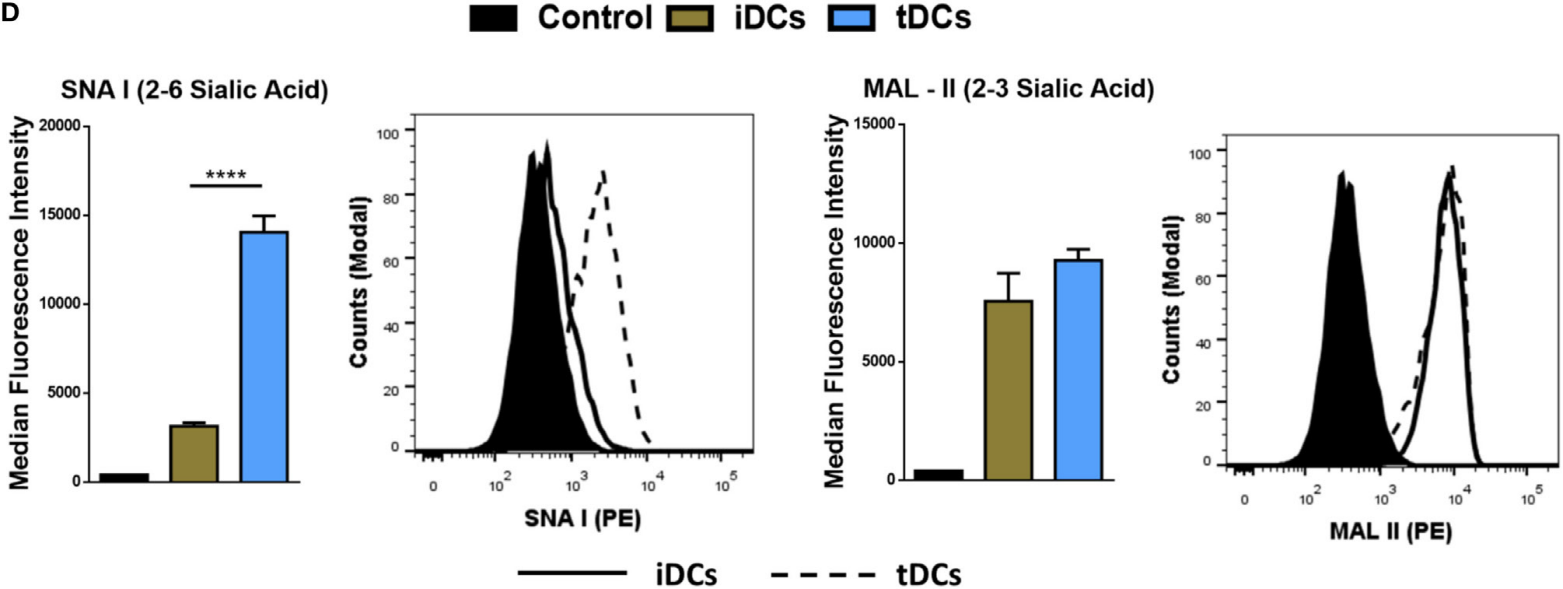
Tolerogenic DC (tDC) generation leads to pronounced changes in glycosylation. **(A)** Unsupervised, hierarchical clustering of previously normalized lectin data (all replicates) was performed with the following parameters: no pre-filtering, complete linkage, Euclidean distance. **(B)** Median responses (*n* = 18) for iDC and tDC glycoproteins at 44 lectins, *p < 0.05, **p < 0.01, ***p < 0.001, ****p < 0.0001. Error bars: median ± average deviation. **(C)** Principal component analysis performed using the 15 lectins, which demonstrated significant signal changes (all *p* < 0.01) and the resulting division of replicate profiles into distinct groups containing predominantly immature DCs (iDCs) (gray border) or tDCs (blue border) with minimal overlap. **(D)** Both iDCs and tDCs were analyzed by flow cytometry for their expression of α2-3-linked Sia (MAL II) and α2-6-linked Sia (SNA-I) (*n* = 3). Streptavidin controls were used for non-specific fluorescence. Representative histograms and bar charts displaying median fluorescence intensity (MFI) for flow cytometric analysis of DC cell surface. Error bars: mean ± SEM *p < 0.05, **p < 0.01, ***p < 0.001, ****p < 0.0001 one-way ANOVA, Tukey’s multiple comparisons test.

Comparisons of all lectin microarray replicate profiles were made by unsupervised hierarchical clustering. This clustering approach revealed two major clusters with separation at 53% minimum similarity (Figure 2A). With the complete linkage method employed, two untreated iDC replicates were placed into the tDC group while only three of the iDC replicates, two from biological set 2 and one from set 5 (Figure 2A), showed outlier behavior and were excluded from the major cluster containing the balance of the iDC replicate data. However, the well-defined separation of the vast majority of the iDC and tDC replicates into two groups (Figure 2A, Group 1 and 2) supports the solidity of the subtle profile differences and also the high level of reproducibility for the lectin profiling method in distinguishing membrane glycoprotein samples from iDCs and tDCs.

Median values obtained from normalized lectin microarray profile data (*n* = 18) for iDCs and tDCs were broadly similar with only small, but significant, differences in intensities noted at a subset of the lectin panel (Figure 2B). The general profiles of tDC glycoproteins remained similar to those of iDCs across lectin features. Furthermore, the lectin profiles displayed no obvious signs of cell stress as evidenced by a lack of elevation of signals suggesting increased endoplasmic reticulum- and proximal Golgi-associated glycan structures (i.e., increased evidence of high mannose structures). However, SNA-I showed a consistent intensity increase with tDC surface glycoproteins (*p* = 2 × 10^−10^) relative to iDCs, which is in line with previous findings from our group (13). PrCA performed using the 15 lectins, which demonstrated *p* < 0.01 (SNA-II, BPA, PNA, DSA, LEL, SNA-I, RCA-I, CPA, ECA, LTA, UEA-I, EEA, GS-I-B4, MPA, and VRA) revealed a division of replicate lectin profiles dominated by distinct groups containing iDCs or tDCs with minimal overlap and further reinforced the ability of these lectins to distinguish untreated iDCs from tDCs (Figure 2C). In short, these lectin microarray profiles demonstrate that the glycocalyxes of the iDC and tDCs are distinct. These changes were validated using lectin-coupled flow cytometry. The increase in SNA-I binding suggests an increase in quantity or better accessibility to α2-6-linked with no significant change suggested for α2-3-linked Sia (MAL-II) confirmed lectin microarray findings (Figure 2D).

### Neuraminidase Treatment of iDCs and tDCs Modulates Levels of α2-6-Linked Sia and Alters Expression Levels of Immunogenicity Markers

**Figure 3:**
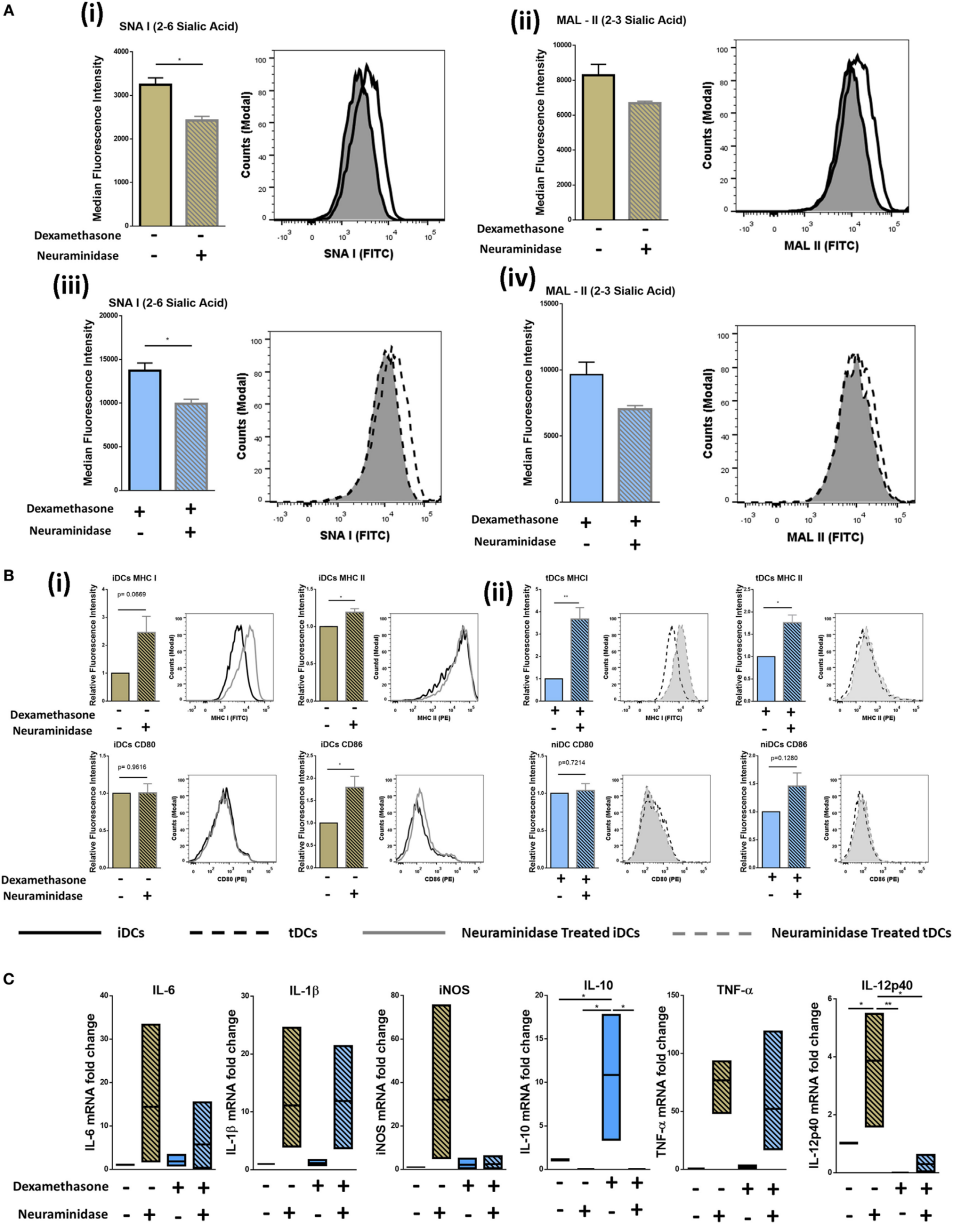
Neuraminidase treatment of immature DCs (iDCs) and tolerogenic DCs (tDCs) decreases levels of α2-6-linked Sia significantly and alters phenotype. iDCs or tDCs were treated with neuraminidase for 90 min at 37°C to cleave Sia residues on the surface. **(A)** α2-6-linked Sia (SNA-I) (i) and α2-3-linked Sia (MAL-II) (ii) was measured on iDCs and niDCs after neuraminidase treatment (*n* = 3). α2-6-linked Sia (SNA-I) (iii) and α2-3-linked Sia (MAL-II) (iv) was measured on tDCs and ntDCs after neuraminidase treatment (*n* = 3). **(B)** Both iDCs (i) and tDCs (ii) were analyzed by flow cytometry for their expression of MHC I (FITC), MHC II (PE), CD 80 (PE), and CD 86 (PE) after neuraminidase treatments. **(C)** The mRNA expression of interleukin 6 (IL-6), interleukin 1 beta (IL-1β), inducible nitric oxide synthase (iNOS), tumor necrosis factor alpha (TNF-α), interleukin subunit beta (IL-12p40), and interleukin 10 (IL-10) was analyzed in iDCs, niDCs, tDCs, and ntDCs. Normalized to GAPDH and fold change relative to iDCs. Representative histograms and bar charts displaying relative fluorescence intensity (RFI) for flow cytometric analysis of DC cell surface. Median fluorescence intensities were established relative to iDCs in the case of niDCs and tDCs in the case of ntDCs. Error bars: mean ± SEM *p < 0.05, **p < 0.01, ***p < 0.001, ****p < 0.0001 one-way ANOVA, Tukey’s multiple comparisons test. Data sets with two groups were analyzed using an unpaired *t*-test.

Sia has long been reported to be important in DC biology (28). Considering the dramatic increase observed after Dexa treatment confirmed by both flow cytometry and lectin microarray (Figures 2B–D), we cleaved Sia using neuraminidase to study phenotypical and functional changes upon removal. iDCs and tDCs were treated with neuraminidase (designated niDC and ntDC, respectively) and lectin binding profiles for SNA-I and MAL-II were analyzed using flow cytometry. Both niDCs and ntDCs showed a significant reduction in SNA-I binding intensities and trend decreases MAL-II binding intensities suggesting the successful removal of α2-6-linked and α2-3-linked Sia, respectively (Figure 3A, i–iv). Based on these results, we further investigated if the removal of Sia resulted in a detectable increase of the expression of MHC I, MHC II, CD80, and CD86 immunogenicity markers after treatment with neuraminidase. niDCs (Figure 3B, i) had small but significant increases in MHC II and CD86 expression when compared to iDCs. MHC I showed a trend increase in expression on niDCs compared to iDCs, and there was no change in CD80 expression after treatment with neuraminidase. ntDCs (Figure 3B, ii) displayed a significant increase in both MHC I and MHC II with no changes in CD80 and a trend increase in CD86 after neuraminidase treatment. niDC and ntDC populations were also assessed for expression of pro- and anti-inflammatory markers by qRT-PCR (Figure 3C). Although there was some sample-to-sample variation, our data indicate that neuraminidase treatment of iDCs leads to dramatic increases in pro-inflammatory mRNA expression of IL-6, IL-1β, iNOS, TNF-α, and IL-12-p40. However, ntDCs are protected from this strong increase in pro-inflammatory cytokine expression in the case of iNOS and IL-12-p40, but mRNA levels of IL-6, IL-1β, and TNF-α are increased. Interestingly, levels of anti-inflammatory IL-10 are lost after neuraminidase treatment in both iDCs and tDCs. In summary, these results indicate that neuraminidase treatment reduces Sia on the cell surface of both iDCs and tDCs and leads to the stimulation of pro-inflammatory cytokine mRNA expression, which can be largely inhibited by Dexa treatment.

### Neuraminidase Treatment Alters Immunomodulatory Properties of iDCs and tDCs

**Figure 4:**
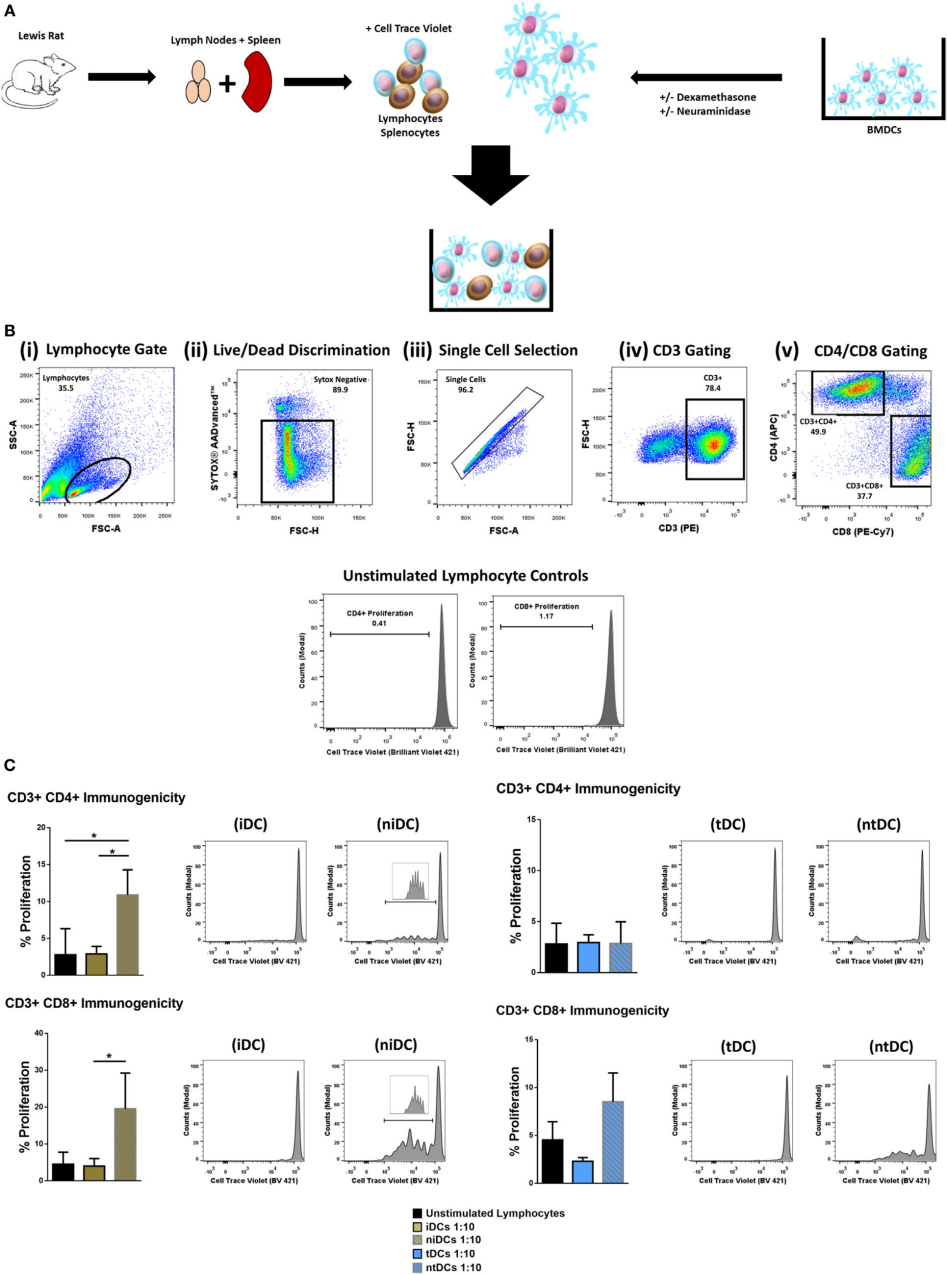
Neuraminidase treatment alters immunogenic properties of immature DCs (iDCs) and tolerogenic DCs (tDCs). To test the immunomodulatory properties of iDCs, niDCs, tDCs, and ntDCs, they were placed into MLRs for 5 days. **(A)** Schematic representation of experimental design. DA iDCs, niDCs, tDCs, and ntDCs were placed in cocultures for 5 days with allogeneic LEW lymphocytes isolated from the spleen and lymph nodes. **(B)** Representative gating strategy. Cells were selected according to size and granularity (i) followed by live/dead discrimination based on Sytox AADvanced^TM^ negative cells (live) (ii). After single cell selection (iii) cells were selected by CD3 (PE) positivity (iv). Further selected by CD4 (APC) and CD8 (PE-CY7) and proliferation was measured by successive generations of CellTrace^TM^ Violet positive cells. **(C)** The ability of iDCs, niDCs, tDCs, and ntDCs to stimulate allogeneic LEW T-cells was analyzed using unstimulated splenocytes/lymphocytes as a negative control (*n* = 3). (i) Representative histograms and bar charts displaying CD4^+^ and CD8^+^ T cell proliferation following a 5-day coculture with iDCs and niDCs. (ii) Representative histograms and bar charts displaying CD4^+^ and CD8^+^ T cell proliferation following a 5-day coculture with tDCs and ntDCs. Error bars: mean ± SEM *p < 0.05, **p < 0.01, ***p < 0.001, ****p < 0.0001 one-way ANOVA, Tukey’s multiple comparisons test.

**Figure 5:**
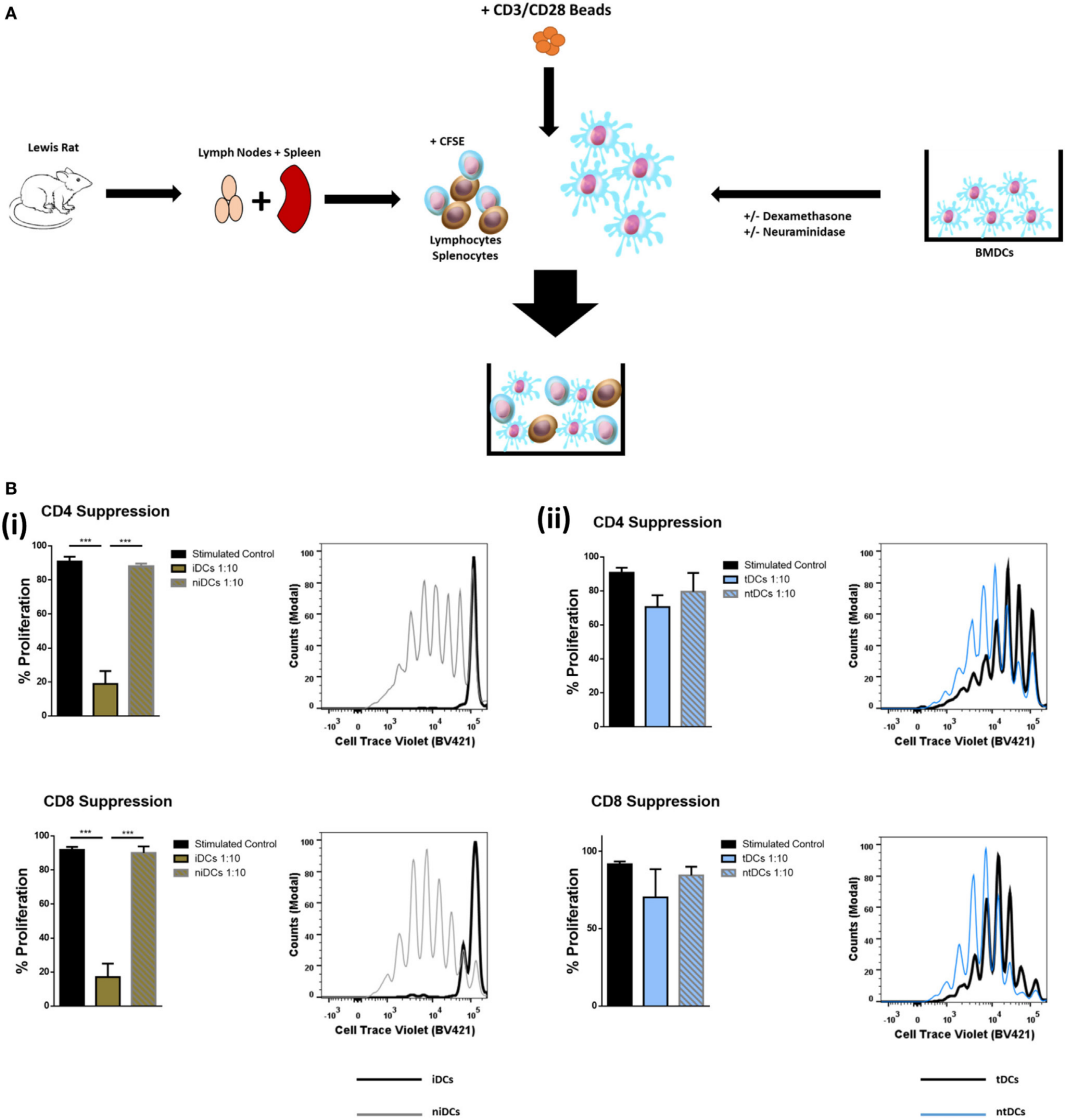
Neuraminidase treatment alters T-cell suppression properties of immature DCs (iDCs) and tolerogenic DCs (tDCs). To test the T-cell suppression properties of iDCs, niDCs, tDCs, and ntDCs, they were placed into stimulated MLR cultures for 4 days. Splenocytes/lymphocytes were stimulated with CD3/CD28 beads. **(A)** Schematic representation of experimental design. DA iDCs, niDCs, tDCs, and ntDCs were placed in cocultures for 4 days with CD3/CD28-stimulated allogeneic LEW lymphocytes isolated from the spleen and lymph nodes. **(B)** The ability of iDCs, niDCs, tDCs, and ntDCs to suppress CD3/CD28 stimulated allogeneic T-cells was analyzed using stimulated splenocytes/lymphocytes as a positive control. Error bars: mean ± SEM *p < 0.05, **p < 0.01, ***p < 0.001, ****p < 0.0001 one-way ANOVA, Tukey’s multiple comparisons test. (i) Representative histograms and bar charts displaying stimulated CD4^+^ and CD8^+^ T cell proliferation following a 4-day coculture with iDCs and niDCs. (ii) Representative histograms and bar charts displaying stimulated CD4^+^ and CD8^+^ T cell proliferation following a 4-day coculture with tDCs and ntDCs.

Considering that the removal of Sia altered the immunogenic phenotype of both iDCs and tDCs, we further analyzed the effects of neuraminidase treatment on iDCs and tDCs through *in vitro* allogeneic coculture experiments. iDCs or tDCs from DA rats were treated with neuraminidase and cocultured with allogeneic lymphocytes. The immunogenic potential or the ability of niDCs and ntDCs to induce the proliferation and/or the activation of allogeneic lymphocytes was analyzed by T-cell proliferation assays (Figure 4A). Responder LEW rat T cells were analyzed based on their co-expression of CD3^+^CD4^+^ or CD3^+^CD8^+^ (Figure 4B). Proliferation of lymphocytes was measured using CellTrace^TM^ Violet (CTV) and activation of lymphocytes was measured using CD25 as an activation marker. DA iDCs (Figure 4C, i) and tDCs (Figure 4C, ii) did not induce an allogeneic response as indicated by a lack of changes in LEW CD3^+^CD4^+^ or CD3^+^CD8^+^ T cell proliferation when compared to unstimulated lymphocytes alone. Additionally, we observed no significant changes in CD3^+^CD4^+^CD25 or CD3^+^CD8^+^CD25 expression (data not shown) supporting our data on reduced immunogenicity of iDCs and tDCs. However, niDCs (Figure 4C, i) significantly stimulated both CD3^+^CD4^+^ and CD3^+^CD8^+^ T cell proliferation when compared to both unstimulated lymphocyte controls and iDCs. This indicates the importance of Sia in the maintenance of an iDCs phenotype. While ntDCs (Figure 4C, ii) show a trend increase to stimulate CD3^+^CD8^+^ T cells, there were no significant changes noted (Figure 4C). To eliminate the possibility of cell death as a potential cause of this increase in proliferation, we assessed cell death using Sytox Blue. We observed that iDCs have less cell death after neuraminidase treatment than tDCs (Figure S2 in Supplementary Material) enabling us to exclude this possibility. Finally, we investigated if niDCs and ntDCs can regulate the proliferation of stimulated T cells. LEW T cells were labeled with CTV, stimulated with CD3/CD28 labeled beads, and cocultured with niDCs and ntDCs (Figure 5A) and CD3^+^CD4^+^ and CD3^+^CD8^+^ proliferation was measured by flow cytometry. Neuraminidase treatment completely abrogates the T cell inhibitory effect of iDCs leading to full restoration of T cell proliferation (Figure 5A, i). Interestingly, Dexa treatment is not sufficient to enable iDCs to inhibit the proliferation of activated T cells as no differences were observed between tDCs and ntDCs (Figure 5B, ii). In summary, these data indicate that the removal of Sia from iDCs increases the immunogenicity by its ability to stimulate CD4 and CD8 T cell proliferation, which can be prevented by Dexa treatment. In contrast, neuraminidase treatment completely restores the proliferation of polyclonally activated T cells, which cannot be prevented by Dexa treatment.

## Discussion

Organ transplantation is often considered as the only therapeutic option for patients with life-threatening organ disease and is now performed on a routine basis. Due to incompatibilities between donor and recipient MHC-molecules, patients are required to take immunosuppressive drugs to prevent the destruction of the transplanted organ by the recipient’s immune system. Immunosuppressive drug regimens are associated with severe side effects long term (34, 35). As a result, alternative immunosuppressive treatment strategies have been researched and developed including the use of therapeutic DCs in the treatment of autoimmune diseases and in the prevention of allograft rejection. DCs promote central and peripheral tolerance through various mechanisms, such as T cell anergy, inhibition of memory T cell responses, and clonal deletion amongst others (36). These characteristics form the basis of the use of DCs in the induction of tolerance. iDCs even have displayed the ability to convert naïve conventional T cells to regulatory T cells (Tregs) both *in vitro* (37, 38) and *in vivo* (39). As shown here, and as shown by others, iDCs in non-inflammatory conditions display a poor immunogenic phenotype. One of the major barriers for use of iDCs in cellular therapies is that they respond to inflammatory stimuli, exemplified here by TLR4 (LPS) stimulation. In the context of autoimmunity and transplantation, iDCs are bound to encounter inflammatory environments if employed in therapeutic regiments. A potential solution to overcome this is the use of tDCs, which are maturation resistant.

Using tDC cellular therapies for the treatment of organ transplantation looks promising (18). tDCs are now routinely generated using different induction protocols, including the use of corticosteroids such as Dexa (11, 14, 15, 17, 40) and, in fact, we have recently shown in a rat model of corneal transplantation that Dexa generated tDCs significantly prolonged allograft survival without the need for additional immunosuppression (13). In this manuscript, we generate tDCs using Dexa and we characterize their maturation resistant phenotype by analyzing the expression of the immunogenicity markers MHCI, MHCII, CD80, and CD86 before and after TLR4 stimulation. We also analyze the expression of several immunomodulatory cytokine mRNAs. Dexa generated, maturation resistant, tDC have been well characterized by us (13, 32) and by other groups (17). However, to our knowledge, little is known on how Dexa induction of tDCs may affect the glycosylation profile of these cells and what functional consequences this may have. Glycosylation changes are not routinely assayed, but are likely to play crucial roles in iDC and tDC biology.

We describe here for the first time, using both lectin microarray and flow cytometry, that generation of tDCs by Dexa treatment leads to significant alterations in the cell surface glycosylation profile when compared to iDCs. We noted highly significant changes in lectin binding for α2-6-linked Sia (SNA-I) with no significant changes in lectin binding for α2-3-linked Sia (MAL-II). Interestingly, Jenner et al. (41) when comparing human iDCs with iDCs matured with a cytokine cocktail (IL-6, IL-1β, TNF-α, and prostaglandin E2) noted decreased α2-6-linked Sia with no changes in α2-3-linked Sia on the more immunogenic DC. This study also showed that Tregs have higher levels of α2-6-linked Sia when compared to activated conventional T cells. This suggests a possible link between α2-6-linked Sia content and tolerogenicity, where the increased α2-6-linked Sia may potentially serve as ligands for inhibitory sialic acid-binding proteins (Siglecs) on the surface of effector cells (41). In fact, hyper-sialylated antigens loaded onto DCs were recently shown to impose a regulatory program in the DCs. This resulted in the inducement of Tregs *via* Siglec-E and the inhibition of effector T cells (42).

Looking more closely at the lectin microarray analysis, other differences in lectin profiles observed here also hint at significant changes in the total abundance or potential branching alterations of underlying oligosaccharide structures, particularly N-acetyllactosamine (LacNAc), which may have occurred because of Dexa treatment. The relationship of responses among the 15 lectins (SNA-II, BPA, PNA, DSA, LEL, SNA-I, RCA-I, CPA, ECA, LTA, UEA-I, EEA, GS-I-B4, MPA, and VRA) which demonstrated the most significant differences between untreated iDCs and tDCs may hold further clues as to the nature of these variations in the glycocalyx, and it is possible that a portion of such variations exist among the membrane glycolipid structures as well as membrane proteins, which were analyzed here. With extracted glycoproteins, only one of the three lectins on the microarray, which has been reported to be indicative of Sia presence, SNA-I, demonstrated a significant intensity increase for tDCs. This was also demonstrated by lectin coupled flow cytometry showing how highly regulated Sia metabolism is in DCs. However, responses at lectins, which bind to structures, which are the most frequent attachment points for sialylation, those which bind to galactose (Gal) or *N*-acetylgalactosamine (GalNAc) (SNA-II, BPA, PNA), and those which bind to the associated disaccharide Type II LacNAc (RCA-I, CPA, ECA) or poly-LacNAc (LEL), are particularly interesting because the expected relationship of higher SNA-I binding and simultaneously lower Gal/GalNAc and LacNAc lectin binding did not consistently hold true across the lectin microarray profiles for DCs. The binding profiles and behavior of SNA-I and MAL-II in these experiments strongly infer quantitative differences between iDC and tDC surface Sia content; however, absolute quantitation will ultimately require chromatographic (e.g., HPLC) or chromatography-conjugated mass spectrometric analysis (LC-MS).

Because of the reported importance of Sias in DC pattern recognition (41, 43), endocytosis/phagocytosis (44–47), antigen presentation (48), migration (28, 49–52), and T cell interactions (28, 53). But also, considering that α2-6-linked Sia was the most significantly increased change after tDC generation by Dexa, we choose to investigate Sia’s importance in iDC and tDC immunogenicity in an allogeneic setting, which would have potential implications in iDC and tDC cellular therapies.

For this, we removed Sia from the surface of the cells by enzymatic digestion using neuraminidase (sialidase). These experiments showed that Sia is involved in maintaining the tolerogenic phenotype of both iDCs and tDCs, as removal of Sia resulted in an increase in immunogenicity markers and increases in pro-inflammatory TH1 mRNA transcripts notably IL-6, IL-1β, iNOS (iDCs only), TNF-α, and IL-12p40 (iDCs only) with significant decreases in anti-inflammatory or tolerogenic IL-10. In experiments where neuraminidase-treated human monocyte derived DCs were cultured with ovalbumin (45) or *Escherichia coli* (44), there were reported increases in immunogenicity markers and cytokine gene expression also. Here, we show that even after Dexa treatment and tDC generation the removal of Sia from the cell surface results in increases in both cell surface immunogenicity markers and TH1 pro-inflammatory cytokine gene expression, underpinning the importance of Sia in a non-immunogenic phenotype.

In the context of allogeneic cell therapy for the treatment of autoimmune diseases and in the prevention of allograft rejection, it is important that the cell therapy itself does not elicit a deleterious immune response. In unstimulated allogeneic co-cultures using LEW responder lymphocytes, we show that iDCs and tDCs are non-immunogentic and do not elicit either CD3^+^CD4^+^ nor CD3^+^CD8^+^ proliferation. This attribute makes them ideal candidates in DC cellular therapies. We show that removal of Sia from iDCs is sufficient enough to stimulate the allogeneic responders, again showing the importance of Sia in a non-immunogenic phenotype. This may indicate that the removal of Sia uncaps underlying structures, which are then recognized as a signal for T-cell proliferation or that the Sias may act as ligands for inhibitory Siglecs on the surface of effector cells and once removed, this inhibitory effect is lost. Sia removal of tDCs did not induce CD3^+^CD4^+^ proliferation, but we noted a trend increase in CD3^+^CD8^+^ proliferation. Interestingly, this indicates that, despite the increase of immunogenicity markers and the transcript increase in several pro-inflammatory mRNAs, Dexa treatment of iDCs was sufficient to keep the cells, at least partially, in a non-immunogenic state.

In CD3/CD28 stimulated (hyper stimulated) allogeneic co-cultures using LEW responder lymphocytes, we show that iDCs had an impressive ability to supress stimulated allogeneic lymphocytes. Sia is critical in maintaining this suppressive ability as when it was absent we observed complete restoration of T cell proliferation for both CD3^+^CD4^+^ and CD3^+^CD8^+^ populations. These results are supported by the fact that Crespo et al. (45) showed increased T-lymphocyte proliferation in autologous mixed lymphocyte cultures using human monocyte-derived DCs where the lymphocytes were stimulated with tetanus toxoid, inactivated with mitomycin C, and cocultured with neuraminidase monocyte-derived DCs. Interestingly, we showed that tDCs do not have the ability to suppress hyperstimulated allogeneic lymphocytes to the same extent as iDCs. Sia removal had little effect on tDCs suppressive ability and did not exaggerate proliferation. Together, these experiments highlight that the tolerogenic properties between iDCs and tDCs are not inherently the same and understanding these characteristics and limitations will inform us on how to optimize therapy strategies.

The findings outlined here could also have numerous implications for our understanding of DC phenotype and function in the tumor microenvironment. Efficient induction of antitumor responses requires that DCs in the tumor undergo proper maturation and activation (54). Understanding DC activation is important both in terms of their role in regulating immune responses locally in the tumor microenvironment (55), and also their use in *ex vivo* cellular and vaccination strategies to induce tumor specific immune responses.

In the context of tumor vaccination strategies using DCs, the required response is to induce tumor-specific effector T cells that can eliminate tumor cells specifically and that can induce immunological memory to control tumor relapse. Our findings suggest that Dexa, a common component of chemotherapy regimens, could suppress DC maturation and activation, their ability to present antigen (56), as well as their ability to induce T cell proliferation and activation. Interestingly, our data indicate that these potent Dexa-induced effects could be somewhat reversed in the presence of a neuraminidase, suggesting a key role for sialylation in Dexa generated tDCs. Removal of sialic acid has also previously been shown to increase tumor antigen-specific T cell responses (48). Our data also show that as well as a more potent ability to induce CD8+ T cell activation. In terms of modulating the tumor microenvironment directly, local delivery targeted approaches using sialyltransferase inhibitors delivered either to the tumor or the local lymph nodes could be exploited. In terms of *ex vivo* generated DCs for either cellular therapy or in vaccination strategies, treatment of DCs with sialyltransferase inhibitors could be sufficient to allow efficient priming of T cells systemically. As DCs provide an essential link between innate and adaptive immunity, these findings could have important implications in our understanding of the suppressive mechanisms within the tumor microenvironment that hinder adaptive antitumor immune responses and potential mechanisms by which they could be overcome.

Together, these results highlight the importance of Sia’s in DC biology, especially in the context of iDC allogeneic cellular therapy. While the precise implications of increased or decreased Sia expression on iDCs and tDCs remain to be elucidated *in vivo*, we show here strong evidence that supports a function of Sia in the therapeutic aspects of DC cellular therapies. Identification of the molecular mechanisms and factors, which are regulated by Sia’s are important to exploit this phenomenon in the clinic. This study points toward the potential of DC surface sialylation as a therapeutic target to improve and diversify DC-based therapies and treatments. In the context of disease, cell glyco-engineering could have positive implications in the treatment of autoimmunity, DC-based vaccines, the tumor microenvironment, and transplant biology.

## Ethics Statement

All animals used in experiments were accommodated in an accredited animal housing facility under a license granted by the Department of Health, Ireland, and were approved by the Animal Ethics Committee of the National University of Ireland, Galway.

## Author Contributions

Substantial contributions to the conception or design of the work (KL, TR, LJ); or the acquisition (KL, OT, HA, PL), analysis (KL, OT, HA, JG, JC), or interpretation of data for the work (KL, JG, AR, TR); and drafting the work or revising it critically for important intellectual content (KL, JG, TR, AR); and final approval of the version to be published (KL, OT, JG, HA, PL, JC, LJ, AR, TR); and agreement to be accountable for all aspects of the work in ensuring that questions related to the accuracy or integrity of any part of the work are appropriately investigated and resolved (KL, OT, JG, HA, PL, JC, LJ, AR, TR).

## Conflict of Interest Statement

The authors declare that the research was conducted in the absence of any commercial or financial relationships that could be construed as a potential conflict of interest. The handling editor declared a past co-authorship with one of the authors TR.

## Supplementary Material

Table S1 Table listing forward primers, reverse primers, and probes for interleukin 6 (IL-6), indoleamine 2,3-dioxygenase (IDO), interleukin 1 beta (IL-1β), inducible nitric oxide synthase (iNOS), and IL-12p40, tumor necrosis factor alpha (TNF-α), GAPDH and interleukin 10 (IL-10).

Table S2 Lectin names and common major binding ligands. Table listing the abbreviations, the kingdom, species, common name, and major binding ligand of the lectins used in lectin micro array profiling.

Figure S1 Isolation and generation of immature DCs and tolerogenic DCs.

Figure S2 Resultant cell death due to neuraminidase treatment of immature DCs (iDCs) and tDCs. Both iDCs and tDCs were treated with neuraminidase for 90 min. These cells were then washed and placed into culture for 48 h. Cells were prepared for flow cytometry as previously described and stained with the live/dead indicator sytox blue (*n* = 1, technical replicate of 2).

## Abbreviations

iDC, immature bone marrow-derived dendritic cell; tDC, tolerogenic bone marrow-derived dendritic cell; niDC, neuraminidase-treated bone marrow-derived immature dendritic cell; ntDC, neuraminidase-treated bone marrow-derived tolerogenic dendritic cell; mDC, mature bone marrow-derived dendritic cell (LPS-treated); MLR, mixed lymphocyte reactions; Sia, sialic acid; Dexa, dexamethasone.
