# Supplementary material for: Regulating Immunogenicity and Tolerogenicity of Bone Marrow-Derived Dendritic Cells through Modulation of Cell Surface Glycosylation by Dexamethasone Treatment
Source: Front Immunol. 2017 Oct 30;8:1427. doi: 10.3389/fimmu.2017.01427 (PMC5670353; doi:10.3389/fimmu.2017.01427)
Supplement: Table S1 — Table listing forward primers, reverse primers, and probes for interleukin 6 (IL-6), indoleamine 2,3-dioxygenase (IDO), interleukin 1 beta (IL-1β), inducible nitric oxide synthase (iNOS), and IL-12p40, tumor necrosis factor alpha (TNF-α), GAPDH and interleukin 10 (IL-10). [file table_1.docx]

**Table 1. RT-PCR Primer design.**

| **Gene** | **Forward** | **Reverse** | **Probe** |
| --- | --- | --- | --- |
| **iNOS** | TTCCCATCGCTCCGCTG | CCGGAGCTGTAGCACGCA | AACACAGTAATGGCCGACCTGATGTTGC |
| **IDO** | CAGGTTACAGCGCCTGGCAC | TCGCAGTAGGGAACAGCAAT | ACATCACCATGGCGTATGTGTGGAA |
| **IL-10** | GAAGACCCTCTGGATACAGCTGC | TGCTCCACTGCCTTGCTTTT | CGCTGTCATCGATTTCTCCCCTGTGA |
| **IL-6** | TCAACTCCATCTGCCCTTCAG | AAGGCAACTGGCTGGAAGTCT | AACAGCTATGAAGTTTCTCTCCGCA |
| **IL-1β** | AACAGCAATGGTCGGGACATA | CATTAGGAATAGTGCAGCCATCTTTA | TTGACTTCACCATGGAACCCGTGTCTT |
| **GAPDH** | NM_017008.4 (Thermo Fisher Scientific, Waltham, MA) |  |  |
| **IL-12p40** | NM_022611.1 (Thermo Fisher Scientific, Waltham, MA) |  |  |
| **TNF-α** | NM_012675.3 (Thermo Fisher Scientific, Waltham, MA) |  |  |
