# Supplementary figures and images for: Regulating Immunogenicity and Tolerogenicity of Bone Marrow-Derived Dendritic Cells through Modulation of Cell Surface Glycosylation by Dexamethasone Treatment
Source: Front Immunol. 2017 Oct 30;8:1427. doi: 10.3389/fimmu.2017.01427 (PMC5670353; doi:10.3389/fimmu.2017.01427)

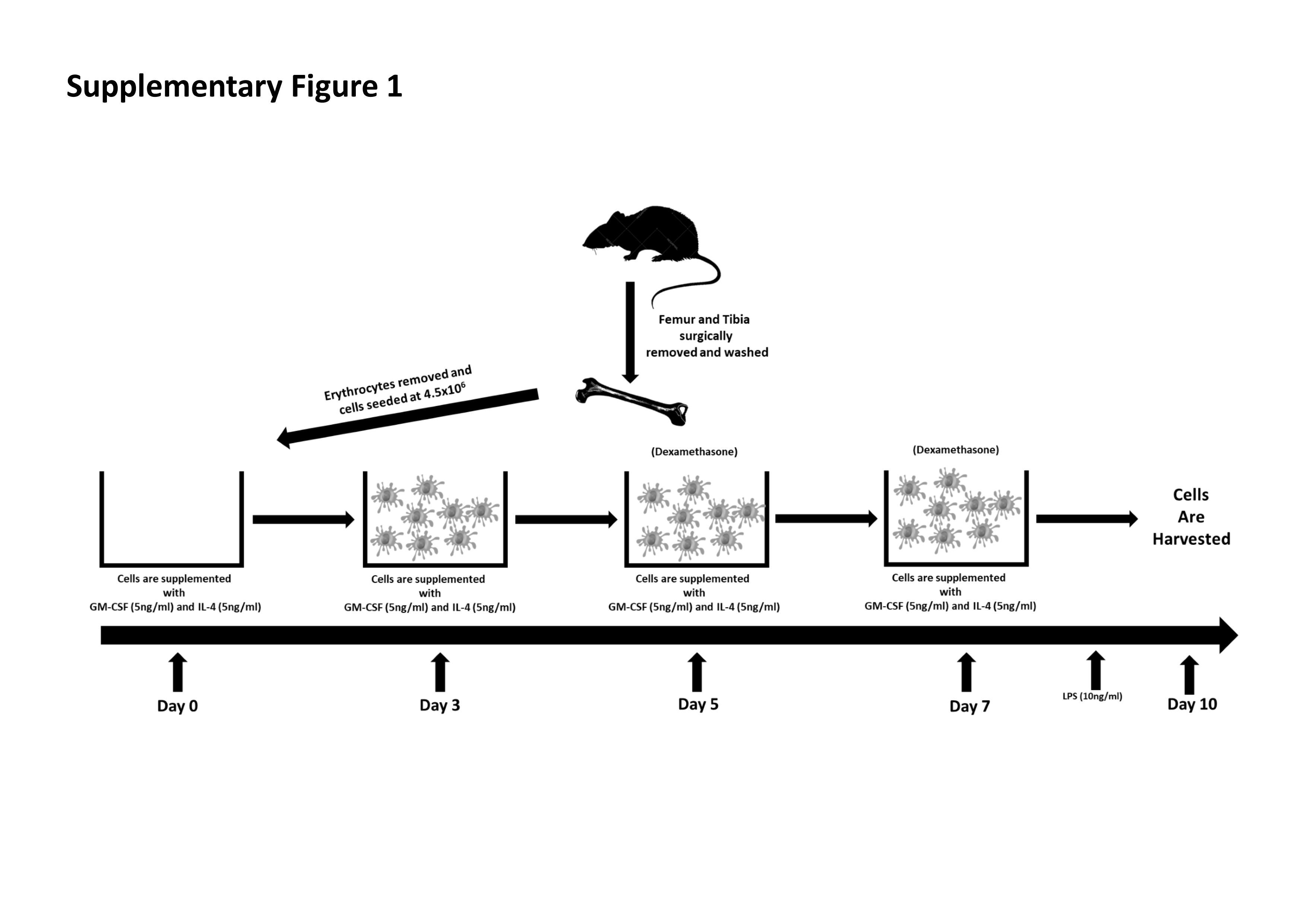

Supplement: Figure S1 — Isolation and generation of immature DCs and tolerogenic DCs. [file image_1.tif]

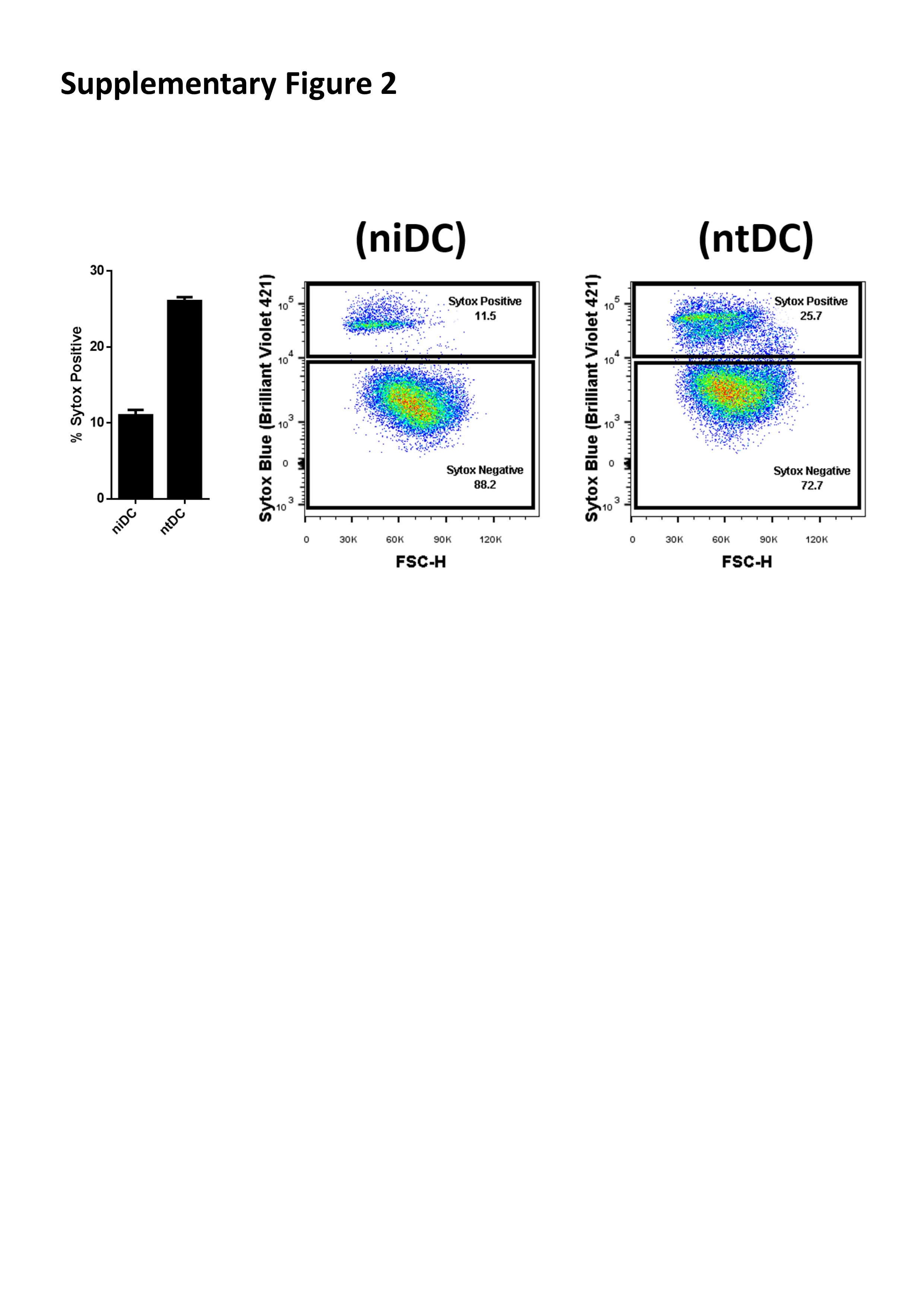

Supplement: Figure S2 — Resultant cell death due to neuraminidase treatment of immature DCs (iDCs) and tDCs. Both iDCs and tDCs were treated with neuraminidase for 90 min. These cells were then washed and placed into culture for 48 h. Cells were prepared for flow cytometry as previously described and stained with the live/dead indicator sytox blue (n = 1, technical replicate of 2). [file image_2.tif]
